# Clinical Lectures on Diseases of the Eye

**Published:** 1864-05

**Authors:** E. L. Holmes

**Affiliations:** Chicago, Lecturer on Diseases of the Eye and Ear in Rush Medical College, and Surgeon of the Chicago Charitable Eye and Ear Infirmary


### CLINICAL LECTURES ON DISEASES OF THE EYE.

By. E. L. HOLMES, M. D., of Chicago,
*Lecturer on Diseases of the Eye and Ear in Rush Medical College, and Surgeon of the Chicago Charitable Eye and Ear Infirmary.*

### THE CORNEA—ACUTE INFLAMMATION.

Gentlemen:—You have seen many cases of inflammation of the cornea, accompanying or following diseases of other tissues of the eye, especially of the conjunctiva. In a large proportion of cases this inflammation—technically called Corneitis or Keratitis—is a secondary disease. It is occasionally, however, idiopathic, and suddenly attacks the cornea of a patient, apparently in good health, in the same way that inflammation, after an exposure to changes of temperature, may attack the lungs or pleura.

Acute Keratitis, to which I wish to call your attention to-day, is far less common than the chronic form of inflammation induced by a scrofulous diathesis. By acute inflammation of the cornea I mean that form of disease which more or less suddenly attacks the whole or a part of the cornea, and terminates within a period of less than four weeks, in recovery or destruction of the parts affected, or becomes chronic. The precise limits between the acute and chronic stages of a disease are not always easily defined, and the division of diseases of the cornea into chronic and acute is perhaps not very practical, and yet, by this division, I think I can best assist you in understanding what you will hereafter read on the subject in larger works.

Acute Keratitis presents the following symptoms, each more or less marked in different cases. There is an opacity of greater or less extent, produced as I have already described in a previous lecture, by a peculiar change in the cells of the cornea. This form of opacity must not be confounded with the opaque cicatrices of former ulcers, which will hereafter be described.

If the disease progresses, the opacity usually becomes either an ulcer or on abscess. I have already informed you how an abscess may become an ulcer. There is pain, which is usually neuralgic in character, not only in the eye but around it; there is often a sensation similar to that produced by a particle of sand under the lids; one of the most constant symptoms is photophobia, with an involuntary tendency to keep the lids closed. The vessels of the conjunctiva and sub-conjunctival tissues are very often congested.

The chief causes of acute corneitis are mechanical injuries, and exposure to changes of temperature. The presence of a scrofulous diathesis, however slight, is a predisposing cause. The pain is sometimes so severe, and the intolerance of light so great, that it is impossible, especially with children, to gain a satisfactory view of the cornea, without the assistance of chloroform. With this agent a careful and thorough examination can be made

In speaking of the treatment of acute corneitis, I shall only call your attention to a few of the leading principles in the therapeutics of three distinct classes of the disease. 1st. Simple inflammation, which does not tend *speedily* to form either ulcers or abscesses. 2d. Ulcerative inflammation. 3d. Purulent inflammation—abscess. Although cases often present so many complications that it is difficult to classify them, still, if you constantly retain in mind a distinct idea of these three groups, you will have little difficulty in comprehending all cases you may meet.

According to my experience, inflammation of the cornea, except in patients of a bad diathesis, is amenable to treatment, although the effects of the disease, in the form of cicatrices, often remain for life.

In the first class of cases, which, possibly with more exactness, may be styled subacute, the patient will complain of more or less indistinctness of vision, with slight photophobia, as shown by the tendency to close the lids in strong lights. An examination will disclose a haziness of the cornea varying in different cases in extent and depth. This marks the limits of the inflammatory process in the cells of the cornea. Sometimes the inflammation subsides, leaving no trace behind. Or it may continue a long time without the formation of ulcers or purulent deposits, and in the form of simple opacity become chronic. In other cases the destruction of tissue either by ulcer or abscess follows at an early period.

In treating these cases it is all important to remove the cause. Unfortunately it is sometimes difficult to discover the ætology since not only individuals are attacked who have received injuries of the cornea, who have suffered from changes of temperature, and who are evidently of an unhealthy diathesis, but also those in whom no such causes can be found. Two of the most abstinate cases of this form of corneitis, I ever observed, were in patients apparently in perfect health.

Everything irritating to the eyes must be avoided. As certain diatheses are strong predisposing causes of corneal affections, it is necessary to correct them by suitable remedies. Nutritious diet, sufficient exercise and pure air are indispensable. In very many cases cod-liver oil, with small doses of iodide of potash, are indicated. Constipation should be overcome and yet care is required in the use of cathartics. Very small doses of calomel or hyd. bichlo. are sometimes beneficial. In many instances nothing is required locally. Usually however, a slightly stimulating collyrium will hasten the ab sorption of the products of inflammation. For this purpose a solution of arg. nit., gr. i—iv to ℥i applied once or twice daily, is suitable. This treatment is usually sufficient in simple cases.

But sometimes from the onset all the symptoms are very violent. The opacity of the cornea becomes rapidly more and more dense, the pain in and around the eye becomes so severe as to prevent sleep, the conjunctiva is congested, and the secretion of tears much increased. It will be observed that the opaque portion of the cornea soon begins to slough. The tissue has lost its vitality and in some respects resembles the slough produced by a moxa. A line of demarkation is formed, the slough, composed of disorganized cells, shreds of intercellular tissue and pus cells, is slowly cast off, leaving an ulcer, which may progress, perforating the cornea, causing a prolapse of the iris, or heal, leaving an opaque cicatrix.

The treatment in this second class of cases must also be directed, as above mentioned, to the general health. The patient should be confined to a room of moderate temperature, and sufficiently shaded. The excruciating pain should be relieved by suitable doses of morphine, and by the local application of atropine as prescribed in a previous lecture. This acts not only as a local anodyne, but also, I believe, as a curative agent in subduing inflammation. If the sensation of heat is a marked symptom the *constant* application of cold compresses is advisable; otherwise I think frequent tepid bathings are preferable. The application of ung. hyd. with ext. belladonna or atropia, is apparently sometimes beneficial. I have often in the West found quinine so beneficial, even where malaria symptoms were not well-defined, that I feel disposed to prescribe it in nearly all these cases. Other Oculists have also found it efficacious. As the symptoms improve, mild astringent collyria are beneficial.

In the third class of cases, those in which the inflammation is confined to the deeper layers of the cornea, tending to produce collections of pus, either in the form of abscess or of onyx, the treatment must be conducted upon the same general principles as in the cases just mentioned. It is remarkable how rapidly the pus of an abscess or onyx may be absorbed spontaneously. As I have already told you, an abscess may sometimes discharge its pus into the anterior chamber and there be absorbed. I advise you to study the application of paracentesis of the cornea. This little operation is performed not only to afford an exit for the pus but also to discharge the aqueous humor, which reduces the pressure upon the cornea, and allays inflammation.

A guarded prognosis should be given in all cases of acute corneitis since vision is often permanently obstructed by opaque cicatrices of the cornea, even where there has been no extensive destruction of tissue.

